# Constitutive activation of glycogen synthase kinase-3β correlates with better prognosis and cyclin-dependent kinase inhibitors in human gastric cancer

**DOI:** 10.1186/1471-230X-10-91

**Published:** 2010-08-12

**Authors:** Yu Jin Cho, Ji Hun Kim, Jiyeon Yoon, Sung Jin Cho, Young San Ko, Jong-Wan Park, Hye Seung Lee, Hee Eun Lee, Woo Ho Kim, Byung Lan Lee

**Affiliations:** 1 Department of Anatomy, Seoul National University College of Medicine, 28 Yeongon-dong, Chongno-gu, Seoul 110-799, Korea; 2 Department of Pathology, Asan Medical Center, University of Ulsan College of Medicine, 388-1 Pungnap-2- dong, Songpa-gu, Seoul 138-736, Korea; 3 Department of Pharmacololgy, Seoul National University College of Medicine, 28 Yeongon-dong, Chongno-gu, Seoul 110-799, Korea; 4 Department of Pathology, Seoul National University Bundang Hospital, 166 Gumi-ro, Bundang-gu, Seongnam-si, Gyeonggi, 463-707 Korea; 5 Department of Pathology, Seoul National University College of Medicine, 28 Yeongon-dong, Chongno-gu, Seoul 110-799, Korea; 6 Cancer Research Institute, Seoul National University College of Medicine, 28 Yeongon-dong, Chongno-gu, Seoul 110-799, Korea; 7 Ischemic/Hypoxic Disease Institute Medical Research Center, Seoul National University College of Medicine, 28 Yeongon-dong, Chongno-gu, Seoul 110-799, Korea

## Abstract

**Background:**

Aberrant regulation of glycogen synthase kinase-3β (GSK-3β) has been implicated in several human cancers; however, it has not been reported in the gastric cancer tissues to date. The present study was performed to determine the expression status of active form of GSK-3β phosphorylated at Tyr^216 ^(pGSK-3β) and its relationship with other tumor-associated proteins in human gastric cancers.

**Methods:**

Immunohistochemistry was performed on tissue array slides containing 281 human gastric carcinoma specimens. In addition, gastric cancer cells were cultured and treated with a GSK-3β inhibitor lithium chloride (LiCl) for immunoblot analysis.

**Results:**

We found that pGSK-3β was expressed in 129 (46%) of 281 cases examined, and was higher in the early-stages of pathologic tumor-node-metastasis (*P *< 0.001). The expression of pGSK-3β inversely correlated with lymphatic invasion (*P *< 0.001) and lymph node metastasis (*P *< 0.001) and correlated with a longer patient survival (*P *< 0.001). In addition, pGSK-3β expression positively correlated with that of p16, p21, p27, p53, APC, PTEN, MGMT, SMAD4, or KAI1 (*P *< 0.05), but not with that of cyclin D1. This was confirmed by immunoblot analysis using SNU-668 gastric cancer cells treated with LiCl.

**Conclusions:**

GSK-3β activation was frequently observed in early-stage gastric carcinoma and was significantly correlated with better prognosis. Thus, these findings suggest that GSK-3β activation is a useful prognostic marker for the early-stage gastric cancer.

## Background

It is thought that human cancers, including gastric carcinoma, develop due to the accumulation of genetic alterations, such as oncogene activation and tumor suppressor gene loss [1-3]. Thus, it is important to identify genetic alterations that affect the behaviors of malignant tumors.

Glycogen synthase kinase-3β (GSK-3β) is a serine/threonine protein kinase whose activity is regulated by site-specific phosphorylation. Complete activation of GSK-3β generally requires phosphorylation at Tyr^216 ^and, conversely, phosphorylation at Ser^9 ^inhibits GSK-3β activity [4]. Although GSK-3β was first described as a component of the metabolic pathway for glycogen synthase regulation, it is now known that GSK-3β is a multi-functional kinase [5]. GSK-3β has more than 40 protein substrates and involved in a wide range of cellular processes, including differentiation, growth, motility and apoptosis [6].

The function of GSK-3β in human cancer cells has been most frequently evaluated in *in vitro *studies, which reported opposing roles of GSK-3β. GSK-3β activation was necessary for proliferation and survival in colorectal cancer cells [7-10], ovarian cancer cells [11] and medullary thyroid cancer cells [12]. In contrast, GSK-3β activation decreased cell proliferation of breast cancer cells [13], prostate cancer cells [14], and colon cancer cells [15] as well as survival of prostate cancer cells [16], breast cancer cells [17], and colorectal cells [8]. Furthermore, *in vivo *xenograft studies also showed inconsistent role of GSK-3β in tumor promotion. Inactivated GSK-3β promoted mammary tumorigenesis [13], whereas activated GSK-3β was essential for tumor growth of skin cancer [18] and medullary thyroid cancer [12]. Regarding the relation between GSK-3β and prognosis in human cancers, there have been few reports. GSK-3β expression has been correlated with a favorable outcome in squamous cell carcinoma of the tongue [19] and breast cancer [20]. In contrast, no significant correlation has been noted between GSK-3β and prognosis in lung cancer [21]. Thus, the biological significance of GSK-3β in each cancer type needs to be elucidated.

Gastric cancer is one of the most common cancers and the major cause of cancer related death worldwide [22]. Thus, a molecular understanding of the genetic factors involved in gastric cancer may contribute toward identifying novel biomarkers. To our knowledge, there are only 2 *in vitro *studies that showed the role of GSK-3β in gastric cancer cells. Mai *et al*. (2006) showed that GSK-3β inhibitors (AR-A014418, SB216763) decreased proliferation and survival of gastric cancer cells [23]. In contrast, Dar *et al*. (2009) observed that GSK-3β suppression increased proliferation of gastric cancer cells [24]. Thus, the role of GSK-3β in gastric cancer cells still remains inconclusive. Recently, Hirakawa et al. (2009) reported the aberrant expression of GSK-3β in surgical gastric cancer samples [25], but they observed only 10 samples, which made it impossible to evaluate the clinicopatholoical significance of GSK-3β in gastric cancer.

In the present study, we extended the previous study to evaluate the expression status of active form of phosphorylated active form GSK-3β (pGSK-3β) in 281 surgically excised human gastric carcinoma tissues using immunohistochemical tissue array analysis. Then, the association between activated pGSK-3β and prognosis, clinicopathological factors or cancer-related molecule was evaluated. In addition, a gastric cancer cell line SNU-668 was treated with a GSK-3β inhibitor lithium chloride (LiCl) to examine the correlation between GSK-3β and cyclin D1 expression.

## Methods

### Patients

The files of 281 surgically resected gastric cancer cases examined at the Department of Pathology, Seoul National University College of Medicine (Seoul, Korea) from January 1 to June 30, 1995 were analyzed. Age, gender, pathologic tumor-node-metastasis (pTNM) stage, lymphatic invasion, lymph node metastasis, and distant metastasis were evaluated by reviewing medical charts and pathological records. The mean age of the patients was 54.8 years, and 93.3% of the patients had undergone curative resection. The cases enrolled in this study included 217 early-stage and 64 late-stage gastric carcinomas. According to the pTNM classification, 77 cases were in stagel, 140 in stage II, 60 in stage III, and 4 in stage IV. Patients who had received preoperative chemotherapy or radiotherapy were excluded from the initial case files. The clinical outcomes of the patients were followed from the date of surgery to either the date of death or to December 31, 2004, resulting in follow-up periods ranging from 1 to 108 months (mean 35 months). Those cases lost to follow-up or those who died of any cause other than gastric cancer were regarded as censored data for the analysis of survival rates.

### Tissue array methods

Six array blocks containing a total of 281 tissue cores obtained from patients with a gastric cancer were prepared by Superbiochips (Seoul, Korea) as described previously [26]. Briefly, core tissue biopsies (2 mm in diameter) were taken from individual paraffin-embedded gastric tumors (donor blocks) and arranged in a new recipient paraffin block (tissue array block) using a trephine apparatus. As reported previously [27], the staining results of the different intratumoral areas of gastric carcinomas in these tissue array blocks showed excellent agreement. A core was chosen from each case for analysis. We defined an adequate case as a tumor that occupied 10% of the core area. Sections (4 μm thick) were cut from each tissue array block, deparaffinized and rehydrated. This protocol was reviewed and approved by the Institutional Review Board of Seoul National University (Approval No. C-0603-162-170).

### Immunohistochemistry

Immunohistochemistry used a streptavidin peroxidase procedure after microwave antigen retrieval. Anti-phospho-GSK-3β^Tyr216 ^(1:50 dilution; BD Transduction Laboratories, San Diego, CA) was used as the primary antibody. Other antibodies were purchased from the following companies: anti-p16^INK4A ^(1:50 dilution) and anti-retinoblastoma protein (pRb) (1:50 dilution) from PharMingen (San Diego, CA); anti-p21^Waf1/Cip1 ^(1:100 dilution), anti-cyclin D1 (1:500 dilution), and anti-adenomatous polyposis coli (APC) (1:400 dilution), anti-SMAD4 (1: 50 dilution) and anti-kangai1(KAI1) (1:200 dilution) from Santa Cruz Biotechnology (Santa Cruz, CA); anti-*O*^6^-methylguanine DNA methyltransferase (MGMT) (1:50 dilution) from Chemicon (Temecula, CA); anti-p27^Kip1 ^(1:100 dilution) from Oncogene (Cambridge, MA); anti-p53 (1:100 dilution) from DAKO Corporation (Carpinteria, CA); anti-PTEN (1:50 dilution) from A.G. Scientific Inc. (San Diego, CA); anti-fragile histidine triad (FHIT) (1:250 dilution) from Zymed (South SanFrancisco, CA); and von Hippel Lindau protein (pVHL) (Prediluted) from Neo Markers (Fremont, CA). Visualization was performed using 3,3'-diaminobenzidine tetrahydrochloride (Sigma, St. Louis, MO).

All immunostained sections were lightly counterstained with Mayer's hematoxylin. To confirm the specificity of each antibody, isotype matched normal serum was used as a negative control. The expression of pGSK-3β in the nucleus and the cytoplasm of gastric tumor cells were considered to exhibit the active form of GSK-3β protein. For cell cycle-related proteins, cancer cells showing nuclear staining regardless of cytoplasmic staining were regarded as expressing the proteins. The results of immunostaining were evaluated by two pathologists (JHK and WHK), who were blinded to the origin of the samples. The kappa value of pGSK-3β expression was 0.816 (*P *< 0.001). For statistical analysis of immunostaining, we initially scored the expression of activated GSK-3β according to the staining intensity as well as the proportion of immunoreactive tumor cells. Then, to show the results clearly, we classify the results into two categories, *i.e.*, positive and negative. In the present study, cases showing immunoreactivity in more than 10% of the tumor cells with more than moderate intensity were considered positive, because it was most reliable cut off value in our previous experiences.

### Cell culture and treatment with a GSK-3β inhibitor

A gastric cancer cell line SNU-668 obtained from the Korean Cell Line Bank (Seoul, Korea) was cultured in RPMI 1640 medium (Life Technologies, Inc., Grand Island, NY) containing 10% fetal bovine serum (Life Technologies, Inc.) and maintained in a 37°C humidified incubator containing 95% air and 5% CO_2_. To inhibit the endogenous GSK-3β activity, cells were treated with 30 mM LiCl for 48 hours and prepared for immunoblotting.

### Immunoblot analysis

Cells were lysed with 1 × Laemmli lysis buffer [2.4 M glycerol, 0.14 M Tris (pH 6.8), 0.21 M sodium dodecyl sulfate, 0.3 mM bromophenol blue] and then boiled for 10 minutes. Protein contents were measured using BCA Protein Assay Reagent (Pierce, Rockford, IL). Samples were diluted with 1 × lysis buffer containing 1.28 M β-mercaptoethanol, and equal amounts of protein were loaded onto 8% SDS-polyacrylamide gels. Proteins were electrophoretically transferred to nitrocellulose membranes, and membranes were then blocked with 7.5% nonfat dry milk in PBS-Tween-20 (0.1%, v/v) at 4°C overnight. They were then incubated with anti-phospho-GSK-3β^Tyr216 ^(1:1000 dilution) from BD Transduction Laboratories (San Diego, CA), anti-cyclin D1(1:1000 dilution) from Cell Signaling Technology (Beverly, MA) or anti-β-actin antibody (1:1000 dilution) from Sigma for 3 hours, and horseradish peroxidase conjugated anti-rabbit or anti-mouse IgG for 1 hour. Immunoreactive proteins were visualized by enhanced chemiluminescence (Amersham, Arlington Heights, IL).

### Statistical analysis

To determine any relationship between the expressions of pGSK-3β and clinicopathological factors or the expressions of other proteins, either the Chi square test or the Fisher's exact test (two-sided) was performed. Survival curves were estimated using the Kaplan-Meier product-limit method, and the significance of differences between the survival curves was determined using the log-rank test. To determine if the expression of pGSK-3β is an independent prognostic variable, multivariate survival analysis was performed using the Cox proportional hazard model. The results were considered statistically significant when *P *values were less than 0.05. All statistical analyses were conducted using SPSS version 12.0 statistical software program (SPSS, Chicago, IL).

## Results

### Expression of activated pGSK-3β

**Figure 1:**
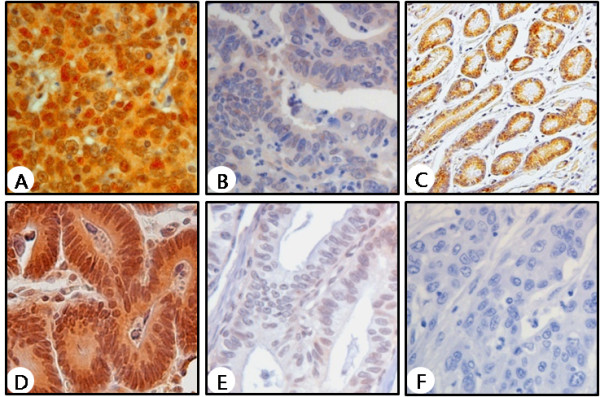
**Representative immunohistochemial features in gastric cancer tissues and normal gastric tissues**. Positive versus negative examples of gastric cancer for pGSK-3β (A and B) and cyclin D1 (D and E) (× 400). (C) A normal gastric tissue stained for pGSK-3β (× 200). (F) Negative control treated without the primary antibodies (× 400).

Figure 1 shows the representative features of immunohistochemical staining of pGSK-3β (Figure 1A-C) and cyclin D1 (Figure 1D and E). The expression of pGSK-3β was shown in the nucleus and the cytoplasm of gastric tumor cells (Figure 1A). In normal gastric tissue, abundant expression of pGSK-3β was observed (Figure 1C). Immunohistochemistry for cyclin D1 showed nuclear immunoreactivity in proliferating gastric tumor cells (Figure 1D). Positive immunoreactivity of pGSK-3β was found in 129 (46%) of 281 surgical gastric cancer specimens (Table 1).

**Table 1:** Correlation between the protein expression of pGSK-3β^Tyr216 ^and clinicopathological factors for 281 human gastric caicinoma specimens.

	Active GSK-3β Positive (%)	Active GSK-3β Negative (%)	*P *value
Total (*n *= 281)	129 (46)	152 (54)	
Age (y)			
0-39	21 (46)	25 (54)	0.881
40-65	83 (47)	94 (53)	
66-99	25 (43)	33 (57)	
Gender			
Male	94 (49)	98 (51)	0.157
Female	35 (39)	54 (61)	
Locus			
Antrum	63 (42)	86 (58)	0.230
Body and Cardia	66 (50)	66 (50)	
WHO classification			
WD	21 (78)	6 (22)	< 0.001*
MD	42 (53)	37 (47)	
PD	44 (37)	74 (63)	
Lauren's classification			
Intestinal	63 (59)	44 (41)	< 0.001*
Diffuse	62 (37)	107 (63)	
Mixed	4 (80)	1 (20)	
TNM stage			
I	53 (69)	24 (31)	< 0.001*
II	60 (43)	80 (57)	
III	16 (27)	44 (73)	
IV	0 (0)	4 (100)	
Lymphatic invasion			
Absent	104 (53)	93 (47)	< 0.001*
Present	25 (30)	59 (70)	
Lymph node metastasis			
Absent	63 (63)	37 (37)	< 0.001*
Present	66 (36)	115 (64)	
Distant metastasis			
Absent	124 (47)	141 (53)	0.304
Present	5 (31)	11 (69)	

WD: well-differentiated adenocarcinoma; MD: moderately-differentiated adenocarcinoma; PD: poorly-differentiated adenocarcinoma.

* Considered to be statistically significant (< 0.05).

### GSK-3β activation and clinicopathological features

Data representing the correlation between activated GSK-3β and the clinicopathologic features of the 281 gastric cancer cases are summarized in Table 1. The expression of pGSK-3β was more prevalent in the well-differentiated type by WHO classification (*P *< 0.001) and in the intestinal type by Lauren's classification (P < 0.001). In addition, the expression of pGSK-3β was more likely to be found in the early pTNM stages (*P *< 0.001). Eighty-eight percent of pGSK-3β positive tumors were in pTNM stages I and II, while 12% were in stages III and IV. Moreover, we found an inverse correlation between the expression of pGSK-3β and lymphatic invasion (*P *< 0.001) or lymph node metastasis (*P *< 0.001). No association was found between pGSK-3β expression and age, gender, tumor location or distant metastasis. These findings indicate that GSK-3β plays an important role in early stages of gastric cancer.

### GSK-3β activation and patient outcome

**Figure 2:**
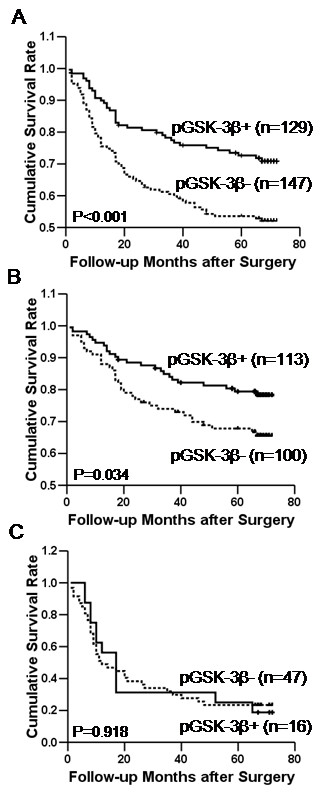
**Kaplan-Meier curves for patient survival**. pGSK-3β-positive carcinomas showed a more favorable prognosis than pGSK-3β-negative carcinomas (*P *< 0.001, A). Survival rate was dependent on pGSK-3β expression in early-stage tumors (*P *= 0.034, B), but not in late-stage tumors (*P *= 0.918, C).

Of the 281 patients evaluated, those with pGSK-3β expression had a significantly higher survival rate than those without pGSK-3β expression (*P *< 0.001, Figure 2A). These findings indicate that pGSK-3β expression has a significant correlation with prognosis of gastric cancer. However, multivariate Cox regression analysis including the pTNM stage revealed that the expression of pGSK-3β was not an independent prognostic factor. In addition, we analyzed the correlation between pGSK-3β expression and patients' prognosis in early-stage tumors (pTNM stages I and II) and late-stage tumors (pTNM stages III and IV) separately, and found that pGSK-3β expression determined the survival rate in early-stage tumors (*P *= 0.034, Figure 2B), but not in late-stage tumors (*P *= 0.918, Figure 2C).

### GSK-3β activation and cell-cycle regulators

Since cell proliferation is mainly regulated by the interaction of various cell cycle regulators, we investigated the relationships between pGSK-3β and cell cycle regulators. Table 2 summarizes the relationship between activation of pGSK-3β and that of the cell cycle regulators in human gastric carcinomas. The expressions of p16, p21, p27 and p53 were statistically significantly higher in pGSK-3β positive gastric carcinomas than in pGSK-3β negative carcinomas (*P *< 0.001, *P *< 0.001, *P *= 0.001, and *P *= 0.013, respectively). In contrast, the expression of cyclin D1 or pRb showed no difference between two groups (*P *= 0.202 and *P *= 0.208, respectively).

**Table 2:** The expression of pGSK-3β^Tyr216 ^in relation to that of cell cycle-regulatory proteins

	Active GSK-3β Positive (%)	Active GSK-3β Negative (%)	*P *value
p16			
Positive	99 (54)	84 (46)	< 0.001*
Negative	24 (28)	63 (72)	
p21			
Positive	38 (26)	39 (74)	< 0.001*
Negative	23 (12)	162 (88)	
p27			
Positive	23 (59)	38 (41)	0.001*
Negative	30 (3)	149 (97)	
p53			
Positive	56 (55)	45 (45)	0.013*
Negative	69 (40)	104 (60)	
pRb			
Positive	118 (45)	142 (55)	0.208
Negative	6 (67)	3 (33)	
Cyclin D1			
Positive	28 (53)	25 (47)	0.202
Negative	90 (43)	119 (57)	

pRb: phospho-retinoblastoma protein.

* Considered to be statistically significant (< 0.05).

### GSK-3β activation and cyclin D1 expression in gastric cancer cells in vitro

**Figure 3:**
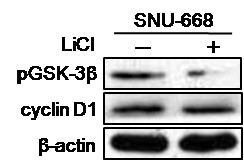
**Protein expressions of pGSK-3β and cyclin D1 in SNU-668 gastric cancer cells**. Cells were treated with or without a GSK-3β inhibitor LiCl (30 mM) for 48 hours and immunoblot analysis was performed for pGSK-3β and cyclin D1. β-actin was used as internal control.

Since the present study showed that there was no correlation between activated GSK-3β and cyclin D1 in clinical gastric cancer samples, we confirmed these data in the *in vitro *experiments using a SNU-668 gastric cancer cell line. Immunoblotting showed that the treatment of SNU-668 cells with LiCl decreased the expression of pGSK-3β, but not that of cyclin D1 protein (Figure 3). These findings suggest that cyclin D1 expression is not regulated by activated GSK-3β.

### GSK-3β activation and other tumor-associated proteins

The correlations between the expression of pGSK-3β and that of tumor suppressor gene proteins are summarized in Table 3. The expression of pGSK-3β positively correlated with that of APC (*P *= 0.002), PTEN (*P *= 0.006), MGMT (*P *< 0.001), SMAD4 (*P *= 0.001), or KAI1 (*P *< 0.002). However, no association was shown between the expression of pGSK-3β and that of pVHL (*P *= 0.091) or FHIT (*P *= 0.387).

**Table 3:** The expression of pGSK-3β^Tyr216 ^in relation to the expression of other tumor suppressor genes

	Active GSK-3β Positive (%)	Active GSK-3β Negative (%)	*P *value
APC			
Positive	102 (52)	94 (48)	0.002*
Negative	21 (30)	48 (70)	
PTEN			
Positive	105 (51)	103 (50)	0.006*
Negative	17 (30)	40 (70)	
MGMT			
Positive	116 (50)	114 (50)	< 0.001*
Negative	10 (25)	30 (75)	
SMAD4			
Positive	117 (49)	121 (51)	0.001*
Negative	7 (19)	30 (81)	
KAI1			
Positive	109 (52)	99 (48)	< 0.001*
Negative	17 (27)	47 (73)	
pVHL			
Positive	118 (47)	131 (53)	0.091
Negative	7 (28)	18 (72)	
FHIT			
Positive	62 (48)	68 (52)	0.387
Negative	56 (42)	77 (58)	

APC: adenomatous polyposis coli; PTEN: phosphatase and tensin homologue deleted on chromosome 10; MGMT: *O*^6^-methylguanine DNA-methyltransferase; KAI1: kangai 1; pVHL: von Hippel Lindau protein; FHIT: fragile histidine triad.

* Considered to be statistically significant (< 0.05)

## Discussion

The assessment of biological prognostic factors is of clinical importance, especially for a disease with poor outcome, such as gastric cancer. Since the biological significance of GSK-3β in gastric carcinoma remains unknown, we used a large scale tissue array-based immunohistochemistry and found that the expression of activated GSK-3β was positively associated with a good prognosis as well as specific clinicopathologic features in 281 human gastric carcinoma samples. We believe this is the first report on the clinical implication of activated GSK-3β in human gastric cancer.

In the present study, we performed immunohistochemical tissue array analysis using an antibody against activated form of GSK-3β (pGSK-3β) and found that activated GSK-3β was expressed in 46% human gastric cancer specimens. pGSK-3β expression was higher in early stage tumors and was inversely associated with lymphatic invasion and lymph node metastasis. Thus, our results suggested that GSK-3β activation is lost during the gastric cancer progression.

There have been a number of conflicting reports concerning the prognostic implication of activated GSK-3β in human cancers. In the present study, we found that GSK-3β activation significantly correlated with favorable prognosis in gastric cancer. Moreover, GSK-3β activation determined the survival rate in early-stage tumors (*P *= 0.034), but not in late-stage tumors. Although pTNM staging is known to have a high prognostic power in gastric cancer, it cannot predict perfectly the outcome for a particular individual. Thus, we think that GSK-3β activation along with pTNM staging may help predict patient outcome more accurately. Our results agreed with previous results shown in squamous cell cancer of the tongue [19] and breast cancer [20]. However, these results do not agree with those in lung cancer [21]. This discrepancy may, at least in part, comes from the differential role of GSK-3β in different cancer cell type as shown in earlier studies [7-14,16-18,20].

Tumor growth is determined by a balance between cell proliferation and apoptosis which are linked by cell cycle-regulatory proteins. Although GSK-3β is known to be a key regulator of numerous signaling pathways, multiple mechanisms are involved in the regulation of cell cycle progression by GSK-3β [28]. The present study analyzed the relationship between GSK-3β activation and cell cycle regulators in gastric cancer specimens. Among the cell cycle regulators, cyclin D1 binds cyclin-dependent kinases (CDKs) and plays critical roles in cell cycle progression through the G1 phase [29]. Although both GSK-3β and cyclin D1 are known to be involved in cell cycle regulation, their relationship in cancer cells remains controversial. Activated GSK-3β has been shown to inhibit cyclin D1 expression in tumor cells including breast cancer cells [30] and squamous cancer cells of the tongue [19]. On the contrary, GSK-3β did not show any correlation with cyclin D1 expression in hepatocellular carcinoma cells [31] and fibroblasts [32]. In the present study, we found that the expression of activated GSK-3β was not correlated with that of cyclin D1 (*P *= 0.202) or pRB (*P *= 0.208) in surgical samples. Furthermore, treatment of gastric cancer cells with LiCl did not change cyclin D1 expression. Thus, our findings suggest that cyclin D1 expression in gastric cancer cells might be regulated by signaling molecules such as Ras, Nuclear factor-κB or β-catenin rather than GSK-3β as previously reported [31,32].

In the present study, activated GSK-3β positively correlated with nuclear expression of CDK inhibitors p16 (*P *< 0.001), p21 (*P *< 0.001), and p27 (*P *= 0.001) as well as that of a tumor suppressor p53 (*P *= 0.013), but not with that of cyclin D1. Since the interplay between CDK inhibitors and cyclin D1-Cdk4 complex is well known, these findings seem paradoxical. However, our results are substantiated by a previous report which showed that treatment of human gastric carcinoma AGS cells with pterostilbene, an active constituent of blueberries, increased the p53, p21, p27, and p16 proteins, whereas the expression of cyclin D1 was not affected [33]. Indeed, a body of evidence accumulated now indicates that cyclin D1 exhibits CDK-independent properties in addition to its CDK-dependent function [29]. To clearly resolve this potential artifact, further molecular studies are needed.

Previously, it was reported that various tumor-suppressive genes were highly expressed in early-stage gastric carcinomas [26]. In the present study, we examined the correlation of activated GSK-3β with those tumor suppressor genes and found that pGSK-3β positively correlated with the expression of p53, APC (*P *= 0.002), PTEN (*P *= 0.006), MGMT (*P *< 0.001), Smad4 (*P *= 0.001), or KAI1 (*P *< 0.002), which were shown to be correlated with good gastric carcinoma prognosis and may act as negative regulators throughout the proliferation or progression of cancer cells [26]. In contrast, correlation was not shown between the expression of pGSK-3β and that of pVHL (*P *= 0.091) or FHIT (*P *= 0.387). The cellular mechanism in relation to the association between activated GSK-3 and tumor suppressive genes in early-stage gastric cancer remains to be elucidated.

## Conclusions

Our results demonstrated that activated GSK-3β was more frequent in the early-stage gastric carcinoma, was negatively associated with nodal status, and was positively associated with better prognosis. In addition, it positively correlated with several tumor suppressor genes, including CDK inhibitors. Although further investigations are needed to clarify the molecular mechanisms involved in GSK-3β activation, the present findings suggest that GSK-3β activation plays an important role probably via inhibition of cell cycle progression, and is a candidate prognostic biomarker in gastric carcinoma.

## Competing interests

The authors declare that they have no competing interests.

## Authors' contributions

JHK has made substantial contributions to conception and design, YJC has made substantial contributions to acquisition of data, and analysis and interpretation of data, as well as has been involved in drafting the manuscript. JY, SJC, and YSK have made substantial contributions to acquisition of data, and analysis of data. JWP, HSL, HEL and WHK have made substantial contributions to analysis and interpretation of data. BLL has been involved in drafting the manuscript and revising it critically for important intellectual content. All authors have given final approval of the version to be published.

## Pre-publication history

The pre-publication history for this paper can be accessed here:

http://www.biomedcentral.com/1471-230X/10/91/prepub
